# Expression of Fbp2, a Newly Discovered Constituent of Memory Formation Mechanisms, Is Regulated by Astrocyte–Neuron Crosstalk

**DOI:** 10.3390/ijms21186903

**Published:** 2020-09-20

**Authors:** Daria Hajka, Przemysław Duda, Olga Wójcicka, Dominika Drulis-Fajdasz, Dariusz Rakus, Agnieszka Gizak

**Affiliations:** Department of Molecular Physiology and Neurobiology, University of Wrocław, Sienkiewicza 21, 50-335 Wrocław, Poland; daria.hajka@uwr.edu.pl (D.H.); przemyslaw.duda@uwr.edu.pl (P.D.); olga.wojcicka@uwr.edu.pl (O.W.); dominika.drulis-fajdasz@uwr.edu.pl (D.D.-F.)

**Keywords:** Fbp2, astrocyte–neuron crosstalk, extracellular vesicles, c-Fos, Hif1α

## Abstract

Fbp2 (muscle isozyme of fructose 1,6-bisphosphatase) is a glyconeogenesis-regulating enzyme and a multifunctional protein indispensable for long-term potentiation (LTP) formation in the hippocampus. Here, we present evidence that expression of Fbp2 in murine hippocampal cell cultures is regulated by crosstalk between neurons and astrocytes. Co-culturing of the two cell types results in a decrease in Fbp2 expression in astrocytes, and its simultaneous increase in neurons, as compared to monocultures. These changes are regulated by paracrine signaling using extracellular vesicle (EV)-packed factors released to the culture medium. It is well accepted that astrocyte–neuron metabolic crosstalk plays a crucial role in shaping neuronal function, and recently we have suggested that Fbp2 is a hub linking neuronal signaling with redox and/or energetic state of brain during the formation of memory traces. Thus, our present results emphasize the importance of astrocyte–neuron crosstalk in the regulation of the cells’ metabolism and synaptic plasticity, and bring us one step closer to a mechanistic understanding of the role of Fbp2 in these processes.

## 1. Introduction

Fbp2 (muscle isozyme of fructose 1,6-bisphosphatase) is a ubiquitously expressed enzyme of glycogen synthesis from non-carbohydrates, e.g., lactate (glyconeogenesis). However, it plays also a variety of non-enzymatic functions. Fbp2 is involved in the regulation of hypoxia-inducible factor 1α (Hif1α) stability, cell cycle-dependent events, biogenesis of mitochondria, and protection of the organelles against high reactive oxygen species (ROS)- and high Ca^2+^-induced stress [1,2,3,4]. This diversity of functions results from the ability of Fbp2 to adopt different oligomeric forms in solution, from monomeric to octameric. In a cell, Fbp2 exists as the mixture of mainly dimers and tetramers. Only the dimeric form of Fbp2 interacts with mitochondria, and only its tetrameric form is retained in the nucleus. The ratio of these oligomeric forms depends on the presence of physiological inhibitors of Fbp2 enzymatic activity—AMP and NAD^+^, which also tetramerize the protein [5].

Recently, we have demonstrated that the presence of Fbp2, presumably its dimeric form, in hippocampal neurons is indispensable for *N*-methyl-D-aspartate receptor (NMDAR)-dependent long-term potentiation (LTP) formation in the hippocampus [6]. LTP, the most studied example of synaptic plasticity, is regarded as the neuronal basis of learning [7]. However, the precise mechanism underlying the persistent synaptic enhancement is not fully understood. It is, for example, widely accepted that astrocytic glycogen degradation followed by lactate transport to neurons (in the process called astrocyte–neuron lactate shuttle) is indispensable for LTP formation [8], but how the astrocytic glycogen-derived lactate stimulates LTP is essentially unknown. Lactate influences the redox state of neurons reducing the NAD^+^/NADH ratio, and it has been shown that such a reduction (or, in other words, elevation of the NADH/NAD^+^ ratio) stimulates activity of NMDAR by modifying the neuronal redox state [9]. However, this also leads to reduction of the pool of tetrameric Fbp2 and increment of the fraction of dimeric Fbp2 in neurons. Dimeric Fbp2, in turn, can interact with neuronal calcium/calmodulin-dependent protein kinase 2 (Camk2), which correlates with Camk2 autophosphorylation. Silencing of Fbp2 expression or simultaneous inhibition and tetramerization of the enzyme in neurons abolishes Camk2 autoactivation and blocks formation of the early phase of LTP and expression of the late-phase LTP markers [6]. We have thus hypothesized that the NAD^+^ level-dependent change in the neuronal Fbp2 dimer/tetramer ratio might be a crucial mechanism by which astrocyte–neuron lactate shuttle regulates LTP formation, making Fbp2 a hub linking neuronal signaling with redox and/or energetic state of brain during the formation of memory traces [6].

Astrocyte–neuron crosstalk incontrovertibly plays a crucial role in shaping neuronal metabolism. It has been shown that it substantially affects the expression of basal metabolic enzymes in both types of cells, by essentially unknown factor(s) released to extracellular space and by cell-to-cell contacts [10]. Although it has been demonstrated that in astrocytes, the expression of regulatory enzymes of glycolysis (phosphofructokinase P and pyruvate kinase M1/M2 isoforms) is stimulated by neuron-derived transthyretin [11], mechanisms regulating the levels of other glucose metabolism enzymes in astrocytes and neurons remain uncharted.

Here, we present evidence that, in murine hippocampal co-cultures of astrocytes and neurons, the Fbp2 protein level is inversely modified by intercellular crosstalk. The presence of astrocytes in the culture elevates the level of Fbp2 protein in neurons and, simultaneously, neurons decrease the level of the enzyme in astrocytes. We show that the observed alterations depend on some factor(s) wrapped in extracellular vesicles (EVs) released to the culture medium.

We also demonstrate that the reduction of the Fbp2 protein level is correlated with changes in Ki-67, c-Fos, and Hif1α protein levels in astrocytes.

The presumable role of Fbp2 in the astrocyte–neuron crosstalk in the context of the regulation of metabolism and synaptic plasticity is discussed.

## 2. Results and Discussion

### 2.1. Fbp2 Expression in Hippocampal Cells Depends on Cell Culture Conditions

To explore the potential regulators of Fbp2 expression in hippocampal astrocytes and neurons, we used murine primary cell cultures and immunofluorescent techniques.

**Figure 1:**
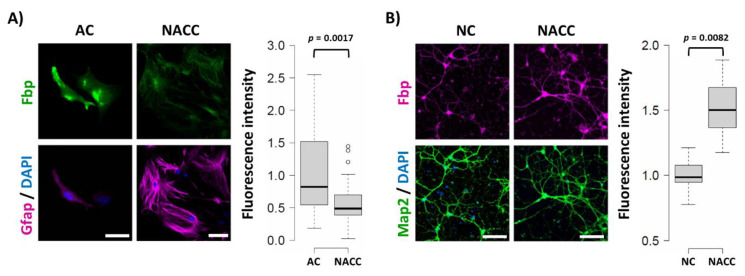
Co-culture-induced changes in the muscle isozyme of fructose 1,6-bisphosphatase (Fbp2) protein-related fluorescence in astrocytes and neurons. (**A**) Co-culturing of neurons and astrocytes (NACC) reduces the Fbp2 protein level in astrocytes (identified by glial fibrillary acidic protein (Gfap) staining) but (**B**) increases it in neurons (identified by microtubule-associated protein 2 (Map2) staining), compared to monocultures of these cells (AC—astrocytic culture; NC—neuronal culture). Box plots present the quantification of the Fbp-related fluorescent signal normalized to control group. Each experiment was repeated at least in triplicate. Bar = 40 µm in (**A**) and 80 µm in (**B**).

We found that the 48 h co-culture of these two cell types led to over 40% reduction of the Fbp2-related fluorescent signal in astrocytes and, simultaneously, to about 50% increase in the signal in neurons, as compared to monocultures of these cells (Figure 1).

### 2.2. Fbp2 Activity and Protein Level Reduction Is Not Reflected by mRNA Changes

**Figure 2:**
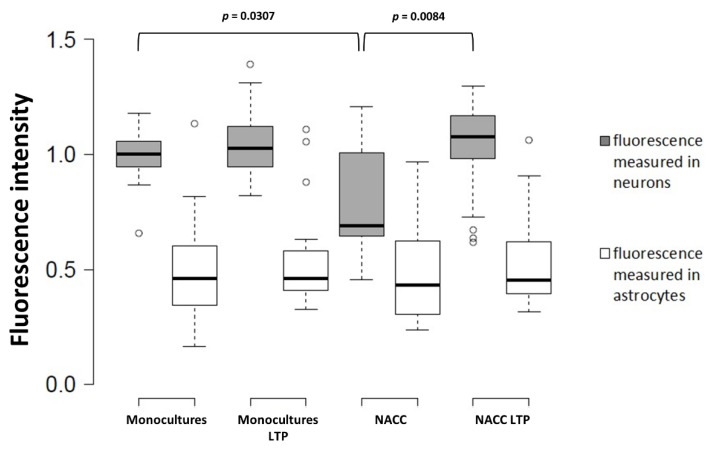
Quantification of the changes in the Fbp2 mRNA level in neuron–astrocyte co-culture under control conditions and after induction of long-term potentiation (LTP). NACC—neuron–astrocyte co-culture, control conditions; NACC LTP—neuron–astrocyte co-culture after induction of LTP. Fluorescence intensity was normalized to control group. The experiment was repeated in triplicate.

Surprisingly, the observed changes in the Fbp2 protein level in astrocytes did not reflect the changes in mRNA quantity, after 48 h of the co-culturing of astrocytes with neurons (Figure 2). Moreover, in neurons co-cultured with astrocytes, the mRNA level for Fbp2 was even lower (~20%) than in neurons from monocultures (Figure 2). This suggests that the reciprocal regulation of Fbp2 level in both types of cells is not related to changes in the expression of the protein but relies on modifications of its stability.

Both neuronal Fbp2 and astrocytes are indispensable parts of neuronal plasticity machinery (LTP) [6,8]; thus, we asked if LTP induction affects the expression of mRNA for Fbp2 in neurons and astrocytes. We found that, in the co-culture, the chemical induction of LTP significantly elevated mRNA for Fbp2 in neurons, but not in astrocytes (Figure 2).

This implies that different mechanisms are responsible for the regulation of Fbp2 levels in these two cell types, under resting conditions and during memory formation. While astrocytes in the co-cultures kept under resting conditions (i.e., without LTP induction) appear to slightly reduce the neuronal amount of mRNA for Fbp2 but increase the stability of the protein, the induction of LTP elevates neuronal expression of mRNA for the enzyme. Under the same resting conditions, neurons decrease the amount of Fbp2 protein in astrocytes by a mechanism independent of mRNA expression, but the induction of synaptic plasticity has no further effect on the Fbp2 level in these cells.

### 2.3. Reduction of Fbp2 Expression Is Correlated with Decrease in the Proliferative Potential of Astrocytes

**Figure 3:**
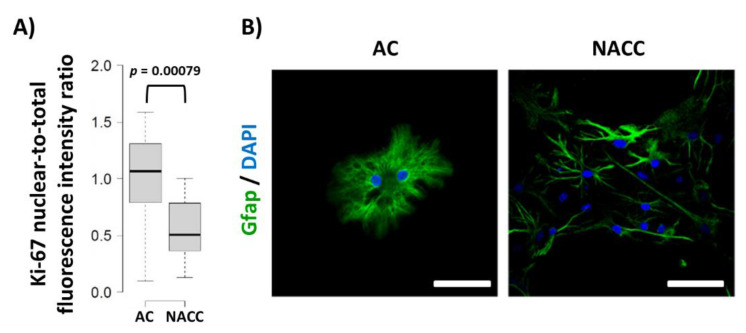
Changes in Ki-67 protein-related nuclear-to-total fluorescence ratio and morphology of astrocytes under different culture conditions. (**A**) Quantification of changes in Ki-67 protein-related fluorescence normalized to control group. (**B**) Morphology of astrocytes stained with anti-Gfap antibodies. DAPI counterstains nuclei. Bar = 50 µm. AC—astrocytic monoculture; NACC—neuron–astrocyte co-culture. The experiment was repeated in triplicate.

We have shown that, in some cell types, Fbp2 is engaged in cell cycle regulation [10]. Thus, we tested the effect of co-culturing on the proliferative potential of astrocytes. It appeared that such a treatment resulted in a significant reduction of Ki-67-related fluorescence in astrocytes as compared to astrocytic monocultures (Figure 3). This was in line with the results of previous experiments on the Ki-67 expression in astrocytes [10,12]. Additionally, we observed changes in the astrocytic morphology from round-shaped to star-shaped, process-bearing (Figure 3), which is in agreement with results of previous studies (our own and other groups) [10,13] showing that neurons affect the morphology of these glial cells as soon as after 24 h of their co-culture.

**Figure 4:**
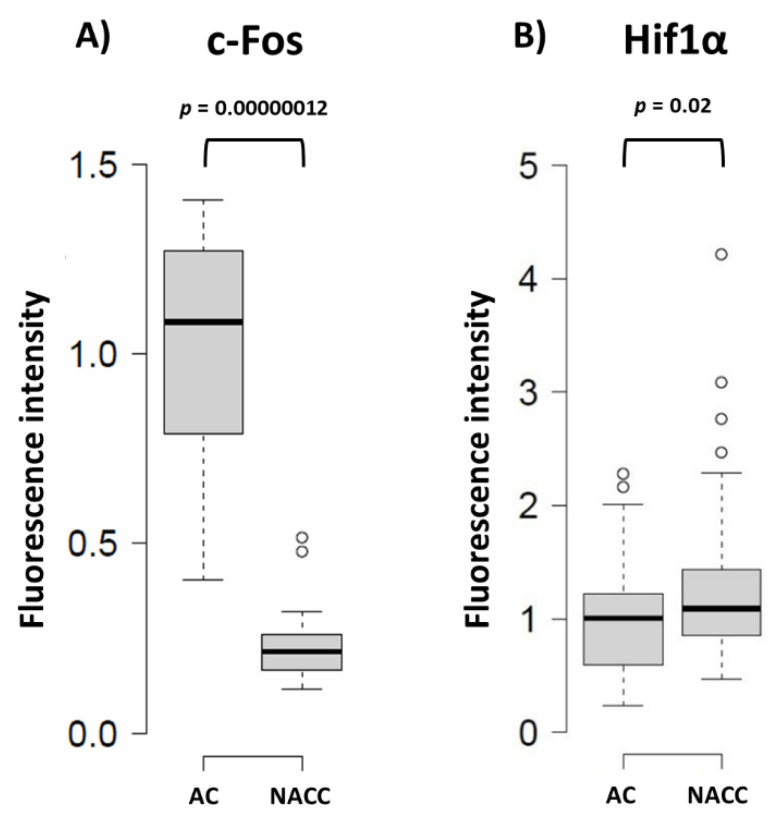
Quantification of the changes in the c-Fos and Hif1α protein-related signal in astrocytes under different conditions. (**A**) Neuron–astrocyte co-culture (NACC) reduces c-Fos-related fluorescence in astrocytes compared to astrocytic monoculture (AC); (**B**) Neuron–astrocyte co-culture increases Hif1α-related fluorescence in astrocytes compared to AC. Fluorescence intensity was normalized to control group.

It has been suggested that proliferation and differentiation of astrocytes are regulated by the c-Fos protein [14]. c-Fos can heterodimerize with c-Jun to form the transcription factor activator protein 1 (AP-1) and control the expression of target genes involved in adaptive responses in the nervous system [15]. Thus, we checked the level of nuclear c-Fos protein in astrocytes, and observed that 48 h of their co-culture with neurons triggered a marked reduction of c-Fos-related fluorescence in this compartment (Figure 4). A similar picture of postnatal maturation-related proliferative potential reduction correlating with Fbp2 expression decrease was recently observed after the addition of cardiac fibroblasts to a monoculture of cardiomyocytes [16].

### 2.4. Reduction of Fbp2 Protein Level in Astrocytes Correlates with Induction of Hif1a Expression and Changes in ROS Production

Previously, it has been shown that Fbp2 and Hif1α protein levels are inversely correlated, because Fbp2 (but also the liver form of fructose 1,6-bisphosphatase, Fbp1) can induce Hif1α degradation [1,17]. Here, we observed that the co-culture of astrocytes and neurons resulting in the decrease in astrocytic Fbp2 titer increased about 20% the overall Hif1α protein-related fluorescence in astrocytes (Figure 4). Not only is Hif1α a major regulator of glycolytic enzymes expression under hypoxic conditions [18], but it can also act as a transcription factor under non-hypoxic conditions (e.g., in astrocytes [19]). Therefore, the Fbp2 reduction-dependent increase in Hif1α protein may be one of the main factors responsible for the observed metabolic reprogramming of astrocytes into glycolytic cells in the presence of neurons [10].

The co-culturing of astrocytes and neurons, similarly to the neuron-conditioned medium (NCM) treatment of astrocytes, resulted in a marked decrease in total cellular ROS production in astrocytes (~70%, *p* < 0.001; Figure S1, Supplementary Materials). Results of studies performed so far have demonstrated that Hif1α reduced mitochondrial ROS production only in cells cultured under hypoxic conditions [20]. In contrast to total cellular ROS, we observed a small but statistically significant (17%, *p* < 0.01; Figure S1) elevation of mitochondrial ROS production under the tested conditions. It is well documented that, in cardiomyocytes and neurons, Fbp2 binds to mitochondria and protects them against Ca^2+^- or ROS-induced excessive depolarization that can lead to swelling of the organelles [1,4,5,6]. Thus, the observed increase in mitochondrial ROS production was not unexpected and reflected a weaker protection of mitochondria resulting from the lowered amount of Fbp2 protein in astrocytes. 

### 2.5. Fbp2 Is Directed to Mitochondria by a Universal, Membrane Depolarization-Related Mechanism But Its Nucleo-Cytoplasmic Shuttling Regulation Is Cell-Specific

**Figure 5:**
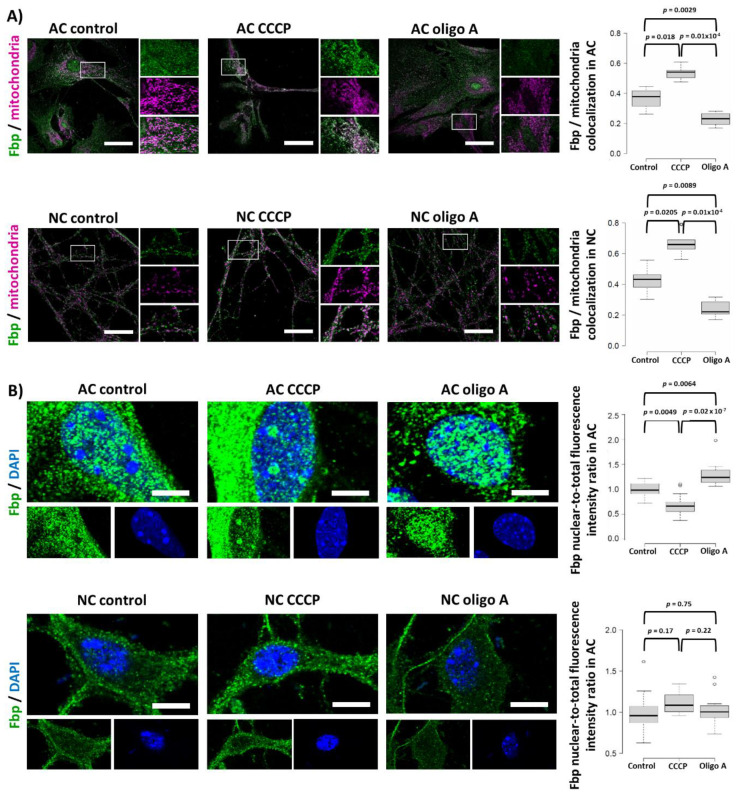
Mitochondrial membrane potential-dependent changes in Fbp2 subcellular localization in astrocytes and neurons. (**A**) Representative microscopic images of Fbp2–mitochondria colocalization and (**B**) nucleo-cytoplasmic distribution of the enzyme together with quantitative analysis of the intensity of Fbp2-related fluorescent signal in these compartments under different treatments are presented. Fluorescence intensity was normalized to control group. AC—astrocytic monoculture; NC—neuronal monoculture; CCCP—carbonyl cyanide m-chlorophenyl hydrazone, the mitochondria-depolarizing agent; oligo A—oligomycin A, the mitochondria-hyperpolarizing agent. (**A**) Bar = 50 µm (AC) or 25 µm (NC); (**B**) Bar = 5 µm. Each experiment was repeated at least in triplicate.

Astrocytes are active participants in synaptic transmission and LTP induction and they respond to neurotransmitters by an increase in intracellular calcium levels. Thus, to test if the mitochondria-protective action of Fbp2 is a universal phenomenon in cells exposed to high [Ca^2+^] or is limited only to “classic” excitable cells (neurons and muscle cells), we examined the effect of mitochondrial membrane depolarizing (carbonyl cyanide m-chlorophenyl hydrazone (CCCP)) and hyperpolarizing (oligomycin A) agents on Fbp2–mitochondria co-localization in astrocytes. As it could be expected based on the Fbp2 behavior in excitable cells, the induction of mitochondrial membrane depolarization resulted in the increase, while hyperpolarization resulted in the decrease in the co-localization of Fbp2- and mitochondria-related fluorescence in astrocytes (Figure 5A). CCCP treatment not only depolarizes mitochondria but it also elevates the level of NAD^+^. In turn, oligomycin A inhibits ATP synthase, which suppresses NADH oxidation. However, because the mitochondrial inner membrane is practically impermeable to NAD^+^, the changes in the NAD^+^/NADH ratio are essentially restricted to these organelles [21,22]. Thus, they are not expected to change the cytoplasmic ratio of NAD^+^/NADH and, hence, the oligomerization status of Fbp2 and its colocalization with mitochondria.

To make sure that the CCCP- and oligomycin A-induced changes in Fbp2 localization are comparable to alterations that we have previously observed in neurons during LTP induction [6], we used these two factors to treat neurons and obtained similar results (Figure 5A).

These results indicate that the decrease in mitochondrial membrane potential (membrane depolarization) is sufficient to stimulate the binding of Fbp2 to mitochondria. Our previous studies, however, identified Ca^2+^ elevation and glycogen synthase kinase 3 (Gsk3) inhibition as the factors inducing Fbp2–mitochondria interactions (e.g., [4]). In a cell, Fbp2 exists as a mixture of (mainly) dimers and tetramers, and only dimers interact with mitochondria [5]. Therefore, it might be inferred that both Gsk3 inhibition and increase in [Ca^2+^] in fact act by elevation of the cellular pool of dimeric Fbp2.

Additionally, in astrocytes, the CCCP/oligomycin A treatment influenced also the nucleo-cytoplasmic ratio of Fbp2 protein-related fluorescence. Conditions stimulating the Fbp2–mitochondria interaction (depolarization) favored withdrawal of Fbp2 from the nucleus, whereas hyperpolarizing conditions increased nuclear retention of the enzyme (Figure 5B). It was not entirely unexpected, as we have shown that factors that induce accumulation of Fbp2 on mitochondria (see above) stimulate also withdrawal of Fbp2 from cells’ nuclei [4].

Previously, we have shown that only tetrameric Fbp2 is retained in the nucleus, as tetramerization occludes the putative nuclear export signal (NES) of Fbp2 [5]. Thus, mechanistically, on the protein structure level, the withdrawal of Fbp2 from nuclei may directly result from dimerization of the enzyme and continuous exposition of its NES. All factors elevating the amount of dimeric Fbp2 and thereby increasing its association with mitochondria simultaneously reduce the population of the tetrameric enzyme and, hence, the nuclear accumulation of Fbp2.

Nuclear Fbp2 was shown to affect c-Myc-dependent mitochondria biogenesis and transcription of oxidative metabolism proteins [3]. Thus, the withdrawal of Fbp2 from astrocytic nuclei seems to be a part of the metabolic reprogramming of astrocytes preparing them, on the one hand, to intensified production of lactate in glycolysis and, on the other hand, to neuron-released glutamate turnover [23] in which mitochondria play a crucial role. Moreover, the cytoplasm-restricted Fbp2 may effectively protect mitochondria against glycolysis-related stress stimuli (such as elevated [Ca^2+^]) despite the reduction of the total amount of the enzyme.

Seemingly surprising was that, in neurons, we did not observe any significant changes in the nucleo-cytoplasmic ratio of Fbp2 after the CCCP/oligomycin A treatment. Instead, Fbp2 was continuously present in this compartment, although the amount of Fbp2 in nuclei (the nuclear-to-total enzyme ratio) was lower than in astrocytes (Figure 5B). However, since neurons rely on oxidative metabolism and Fbp2 therefore regulates c-Myc-dependent transcription, the constant presence of Fbp2 in neuronal nuclei may be required to precisely adjust oxidative metabolism allowing for relatively fast changes in the expression of mitochondrial proteins.

In turn, the observed differences in the amount of nuclear Fbp2 might be related to a phase of cell cycle, as we have shown that Fbp2 accumulates in nuclei in the S phase of the cycle [2] and that neurons, in contrast to astrocytes, are in the post-mitotic G0 phase, and they re-enter the S phase only in a pathological situation such as neurodegeneration [24].

Together, these results suggest that although Fbp2 is directed to mitochondria by a universal, membrane depolarization-related mechanism, the nucleo-cytoplasmic shuttling of the protein is regulated in a cell-specific manner.

### 2.6. Fbp2 Protein Level in Hippocampal Cells Is Regulated by Extracellular Vesicle Cargo

Next, we decided to test if the changes in the Fbp2 protein level resulted from astrocyte–neuron physical interactions or were chemical factor-mediated. Since neuronal cell cultures poorly tolerate the exchange of their culture medium, we focused mainly on astrocytes.

**Figure 6:**
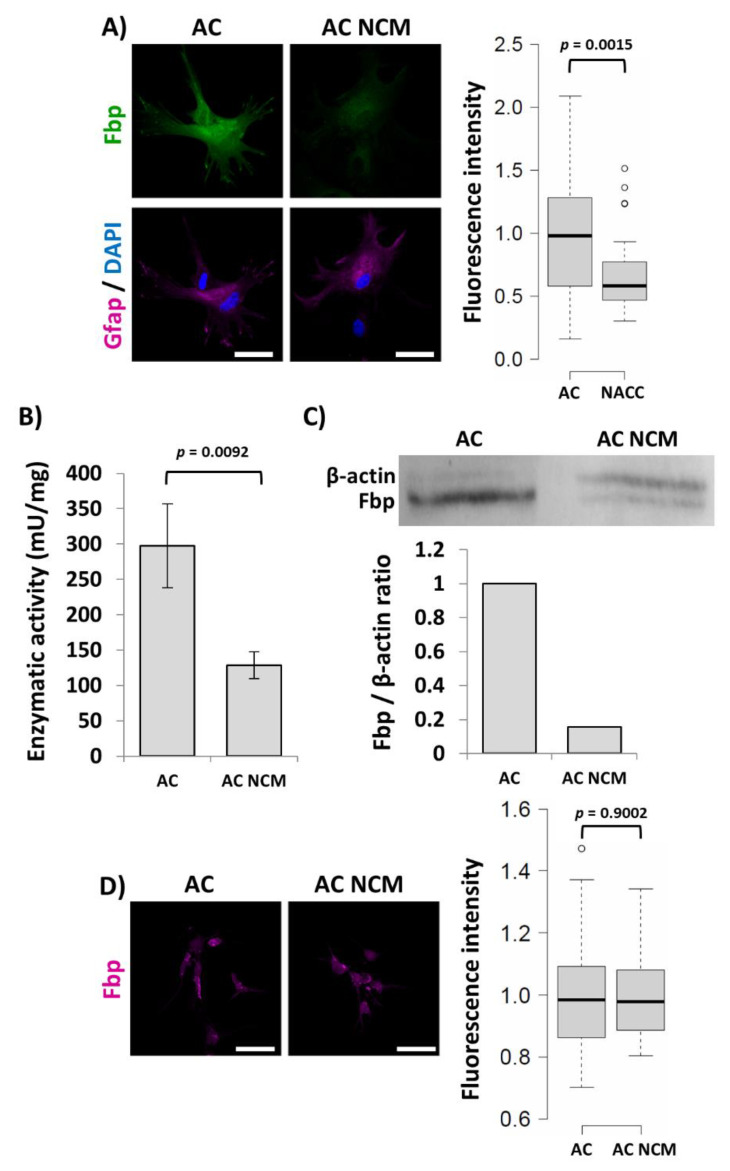
The changes in Fbp2 protein but not mRNA level in astrocytes induced by their co-culturing with neurons can be reproduced by the culturing of astrocytes in neuron-conditioned medium (NCM). (**A**) NCM reduces the level of Fbp2-protein-related fluorescence in astrocytes. The graph presents the quantification of the Fbp-related immunofluorescent signal. (**B**) NCM reduces the enzymatic activity of Fbp2 in homogenates of astrocytic monocultures. Results are expressed as a mean and standard deviation. (**C**) Western blot (WB) membrane and quantification of Fbp-related staining in reference to the β-actin staining. (**D**) Confocal image and quantification of the fluorescent signal related to mRNA for Fbp2. AC—astrocytic monoculture; AC NCM—astrocytic monoculture treated with NCM. Bar = 40 µm. Each fluorescence-based experiment was repeated at least in triplicate.

We observed that the effect of the co-culturing on Fbp2-related fluorescence in astrocytes was easily reproduced by 48 h incubation of astrocytic monocultures with neuron-conditioned medium (NCM), resulting in over 30% reduction of the fluorescence (Figure 6A). Incubation of astrocytes in the fresh neuronal medium even increased the Fbp2-related fluorescence making the decrease in the signal after the NCM treatment even more pronounced.

Additionally, the NCM treatment led to about 70% decrease in the enzymatic activity of Fbp2 in homogenates from astrocytes, compared to control conditions (i.e., cells cultured in astrocytic medium; Figure 6B) and reduction of the Fbp2 protein amount, as determined by densitometric analysis of Western blot (WB) (Figure 6C). Thus, it might be concluded that neurons regulate the astrocytic Fbp2 level by some factor(s) released to the cell culture medium.

Consistently with the results of co-culturing, the NCM treatment-induced changes in the Fbp2 protein level in astrocytes did not reflect the changes in mRNA quantity; after 48 h of the treatment, the level of mRNA for Fbp2 was practically unchanged in astrocytes (Figure 6D).

Searching for factors that could trigger the observed changes in the Fbp2 protein level in astrocytes, we found that the frozen–thawed neuron-conditioned medium did not have any effect on the parameter. Thus, we assumed that the sought factor(s) could not cross the cell membrane directly but must be contained in extracellular vesicles that are released to the culture medium but destroyed by the freeze–thaw process. It has been demonstrated that neurons as well as glial cells release extracellular vesicles (EVs) of different kinds, both under physiological and pathological conditions (for a review, see [25]).

**Figure 7:**
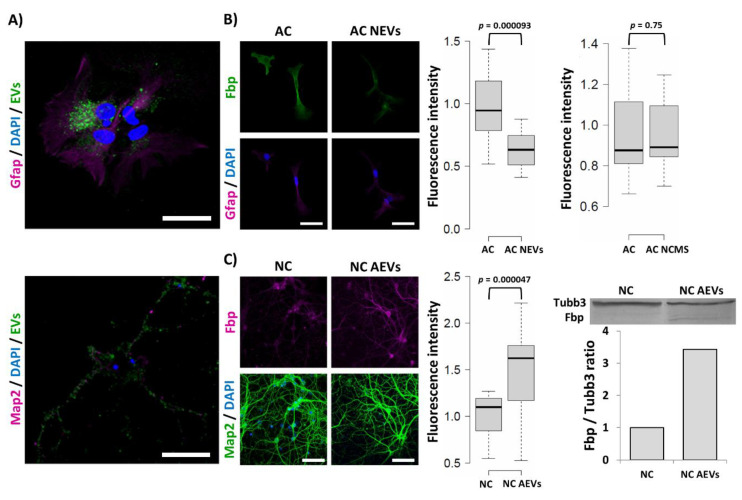
Extracellular vesicle-induced changes in the Fbp2 protein level in astrocytic and neuronal monocultures. (**A**) PKH67-stained (green) extracellular vesicles (EVs) absorbed by astrocytes (counterstained with Gfap) and neurons (counterstained with Map2). Bar = 40 µm. (**B**) Isolated neuronal EVs (NEVs) but not the EV-depleted supernatant (neuronal culture medium supernatant - NCMS) reduced the Fbp2 protein level in astrocytic cultures (ACs). Bar = 50 µm. (**C**) Isolated astrocytic EVs (AEVs) elevated the Fbp2 protein level in neuronal cultures (NCs), which was confirmed by Western blot. The WB membrane and densitometric analysis of Fbp-related band in reference to tubulin β3 (Tubb3) band is presented. Bar = 80 µm. Each fluorescence-based experiment was repeated at least in triplicate.

To test our hypothesis, the neuron-conditioned medium was “fractionated” into supernatant and vesicular fractions using ultracentrifugation [16]. Then, the EVs were stained with a green fluorescent dye PKH67 and added to monocultures of astrocytes (Figure 7). Alternatively, astrocytic monocultures were incubated with the unstained vesicles or supernatant fraction and the Fbp2-related fluorescence was quantified. Treatment of the cells with isolated EVs mimicked the effect of the neuron–astrocyte co-culture on the Fbp2-related signal. In turn, the conditioned medium depleted of the vesicle fraction (supernatant) was unable to induce any changes (Figure 7).

Moreover, the addition of EVs isolated from astrocyte-conditioned medium to neuronal monoculture was sufficient to increase Fbp2-related fluorescence in neurons, to a similar level as in the co-culture, which was confirmed by Western blot of homogenates from astrocytic EV-treated neurons (Figure 7).

To summarize, here, we present evidence that in murine hippocampal co-cultures of astrocytes and neurons, expression of Fbp2 is inversely modified by intercellular crosstalk. The presence of astrocytes in the culture elevates the level of Fbp2 in neurons and, simultaneously, neurons decrease the level of the enzyme in astrocytes. We show that the observed shift is regulated by paracrine signaling using yet unidentified factor(s) packed in extracellular vesicles released to the culture medium by astrocytes and neurons.

The changes in the amount of Fbp2 protein evoked by the astrocyte–neuron crosstalk are not related to alterations in the rate of the Fbp2 mRNA expression but they result from the regulation of protein stability/degradation. However, the induction of excitatory plasticity (LTP) stimulates expression of the Fbp2 mRNA in neurons but does not change it in astrocytes.

The alteration of the Fbp2 protein level might be related to metabolic coupling of the two cell types and simply reflect the intensification of glyconeogenesis in neurons co-cultured with astrocytes; however, most studies do not support the hypothesis that neurons may synthesize glycogen from carbohydrate precursors. Moreover, in the light of all previous findings, it is conceivable that if non-enzymatic functions of Fbp2 are required for LTP formation, there is also a “non-enzymatic” reason of observed changes in the expression of the enzyme in neuron–astrocyte co-cultures. Thus, the astrocyte-mediated increase in neuronal Fbp2 protein seems to be a mechanism preparing neurons for induction of NMDAR-dependent plasticity.

The reduction of the Fbp2 protein level in astrocytes caused by neurons is likely to be a part of the mechanism by which neurons rearrange astrocytic physiology to support neuronal transmission and some forms of plasticity (e.g., the NMDAR-dependent LTP). It is known that astrocytes co-cultured with neurons exhibit glycolytic metabolism and more mature morphology than astrocytes growing in the absence of neurons or neuron-derived factors [10,26]. In this report, we show that the reduction of the Fbp2 level results in a decrease in c-Fos—a factor engaged in astrocyte proliferation and differentiation [14]. Importantly, the neuron-dependent reduction of the Fbp2 amount in astrocytes results in the elevation of the Hif1α protein level, thereby stimulating the expression of glycolytic proteins [18] and, also in the Hif1α-dependent manner, curbing mitochondrial oxidation [20]. Furthermore, since Fbp2 is indispensable for the regulation of c-Myc-dependent mitochondria biogenesis [3], the neuron-dependent reduction of the Fbp2 level should affect both the oxidative capacity of astrocytes and the neurotransmitter clearance.

Thus, our present results emphasize the importance of astrocyte–neuron crosstalk in the regulation of the cells’ metabolism and synaptic plasticity, and bring us one step closer to a mechanistic understanding of the role of Fbp2 in these processes.

## 3. Materials and Methods

### 3.1. Cell Culture and Treatment

Cells were cultured at 37 °C in a humidified atmosphere with 5% CO_2_. Hippocampal neurons were isolated from newborn BALB/c mice and cultured as described previously [27] with a minor modification—the glucose concentration in the culture medium was 2.5 mM. All the experiments were performed on 14–16-day-old neuronal cultures (NCs). Hippocampal astrocytes were isolated from newborn BALB/c mice and cultured (ACs—astrocytic cultures) as described in [10]. To obtain the astrocyte–neuron co-cultures (ANCCs), astrocytes (40,000/cm^2^) were seeded into 14-day-old neuronal cultures. The experiments were performed after 48 h of the co-culturing.

To determine if neurons affect the expression of astrocytic proteins by releasing some molecules into the culture medium, astrocytic monocultures were incubated for 48 h in the medium collected from 14–16-day-old neuronal culture (neuron-conditioned medium, NCM). In control experiments, the cells were cultured in an astrocytic or fresh neuronal medium.

LTP was induced according to the protocol described previously [28,29]. In brief, 2-week-old neuronal cultures were transferred to the Ringer’s solution (137 mM NaCl, 5 mM KCl, 1.89 mM CaCl_2_, 10 mM HEPES, 33 mM glucose, pH 7.3, 37 °C) and LTP was induced with 1 µM strychnine and 200 µM glycine. The induction time was 60 min, after which the cultures were fixed. The presence of potentiation was not recorded.

To hyperpolarize or depolarize mitochondrial membrane, oligomycin A (10 µM) or carbonyl cyanide m-chlorophenyl hydrazone (CCCP, 50 µM) was used, respectively [30]. The cell cultures were transferred to the Ringer’s solution (37 °C) and incubated with the chemicals for 5 min. Afterward, the cells were fixed.

### 3.2. Immunofluorescence

The immunofluorescence studies were performed as described previously [10]. The cells were incubated overnight at 4 °C with respective primary antibodies: rabbit anti-Fbp (1:400, isolated, purified, and validated as described in [31]), mouse anti-Fbp (1:500, Santa Cruz Biotechnology, Dallas, TX, USA, sc-271799), rabbit anti-Ki-67 (1:300, Abcam, Cambridge, UK, ab15580), rabbit anti-Tomm20 (1:1000, Merck KGaA, Darmstadt, Germany, HPA011562), mouse anti-c-Fos (1:1000, Merck KGaA, Darmstadt, Germany, 2G9C3), mouse anti-Hif1α (1:300, Merck KGaA, Darmstadt, Germany, SAB5200017), mouse anti-Gfap (1:500, Merck KGaA, Darmstadt, Germany, G3893), rabbit anti-Gfap (1:500, Merck KGaA, Darmstadt, Germany, G926), and mouse anti-Map2 (1:500, Merck KGaA, Darmstadt, Germany, N9942). The primary antibodies were detected using fluorophore-labeled secondary antibodies: goat anti-rabbit-AlexaFluor488 (1:500, ThermoFisher Scientific, Waltham, MA, USA, A11034), goat anti-mouse-AlexaFluor633 (1:500, ThermoFisher Scientific, Waltham, MA, USA, A21050), goat anti-rabbit FITC (1:800, Merck KGaA, Darmstadt, Germany, F6005), and goat anti-mouse TRITC (1:150, Merck KGaA, Darmstadt, Germany, T7782). Nuclei were counterstained with DAPI.

### 3.3. ROS Visualization

Total ROS levels were detected with 2′,7′-dichlorofluorescin diacetate (DCFDA; Merck KGaA, Darmstadt, Germany, D6883). The cultured cells were washed once with warm HBSS and incubated for 30 min with 10 μM DCFDA in HBSS in the cell culture incubator (Forma^TM^ Steri-Cycle^TM^ i160, ThermoFischer Scientific, Waltham, MA, USA). The fluorescence yield level, which is identical to the intracellular ROS content, was observed using an OLYMPUS IX71 fluorescence microscope at Ex/Em = 495/519 nm and expressed as relative fluorescence units using the ImageJ software [32].

To visualize mitochondrial ROS production, cells were incubated with 0.5 μM MitoTracker Red CM-H2XRos (ThermoFisher Scientific, Waltham, MA, USA, M7513) instead of DCFDA, and examined after fixation (Ex/E = 579⁄599 nm).

### 3.4. Fluorescence In Situ Hybridization (FISH)

FISH was performed as described previously [10]. The following oligonucleotides complementary to mouse mRNA sequences were used: Fbp2 (5′-(Cyanine3)GCACACAGCT GAGATACTCT TGCACATCCT CAGGGGAC-3′). Oligonucleotides were synthesized by Merck KGaA. In controls, the oligonucleotide probes were omitted.

### 3.5. Isolation and Visualization of Extracellular Vesicles

The culture media from astrocytic and neuronal monocultures were collected and the vesicular fraction was isolated as described in [33]. To reduce contamination with vesicles derived from animal serum, the Exosome-Depleted Fetal Bovine Serum Qualified One Shot (ThermoFischer Scientific, Waltham, MA, USA) was used instead of FBS in astrocytic culture medium. B27 Supplement used instead of FBS in neuronal culture should not be contaminated by any vesicles. To confirm isolation and uptake of the vesicles by cells, the vesicles were stained with PKH67 Green Fluorescent Cell Linker Kit (Merck KGaA, Darmstadt, Germany), incubated with cell cultures and visualized using confocal microscopy as described before [16]. Alternatively, the cell cultures were treated with unstained vesicles or supernatant that remained after the isolation procedure.

### 3.6. Confocal Microscopy

Confocal microscopy analysis was performed as described previously [10] with an Olympus FV1000 confocal microscope using Plan Apo 60×/1.35 NA Oil and 40× UPlanSApo 40×/0.95 NA objectives. At least 15 images of randomly selected areas (edges of slides were avoided) were taken from each microscopic slide. The quantification of the fluorescence was performed using ImageJ software [32]. For the analysis of protein or mRNA expression, the mean fluorescence intensity was measured from regions of interest (ROIs). The ROIs were defined based on the Map2 or Gfap immunostaining, which delineates the neuronal and astrocytic cells, respectively. To establish a nuclear localization of proteins, the protein-associated fluorescence from the nucleus (DAPI) area was measured and expressed as a ratio to the total protein-associated fluorescence from the same cell.

In all the experiments using confocal microscopy, 30 to 60 measurements were taken from each of the cell culture conditions. For the analysis of Fbp–mitochondria colocalization, the Mander’s coefficient was used as described in [6]. Each experiment was repeated at least in triplicate.

### 3.7. Fbp2 Activity Measurement

For this study, 90% confluent astrocytic cell cultures were dissociated with trypsin and centrifuged and cell pellets were mechanically homogenized in a homogenization buffer (50 mM Tris, 10 mM EDTA, 10 mM EGTA, 60 mM NaF, 0,5 M sucrose) with the addition of a Protease Inhibitors Set (Merck KGaA, Darmstadt, Germany, 11206893001). The homogenates were centrifuged at 15,000× *g* for 20 min in 4 °C to remove cell debris. Fbp2 activity was measured as described in [34], in triplicate. Briefly, Fbp2 enzymatic activity, expressed in U (µmol∙min^−1^), was determined spectrophotometrically from the difference in the slope of NAD(P)H absorbance (340 nm; ε = 6.22 mM^−1^∙cm^−1^) before and after the addition of the substrate (fructose-1,6-bisphosphate). The activity was measured in 50 mM Bis-Tris Propane buffer with 150 mM KCl, 0.25 mM EDTA, and 5.25 mM MgCl_2_, pH 7.4, 37 °C. The content of the reaction mixture (V = 1 mL) was as follows: 0.2 mM NADP, 2 units of glucose-6-phosphate dehydrogenase, 2 units of phosphoglucose isomerase, and 0.2 mM fructose-1,6-bisphosphate. Spectrophotometric measurements were performed with the Agilent 8453 diode array spectrophotometer (Agilent Technologies, Santa Clara, CA, USA). Results are expressed as a mean and standard deviation.

### 3.8. Western Blot

Western blot of astrocytic protein extracts was performed as described in [35] using the following antibodies: rabbit anti-Fbp (1:1000, isolated, purified, and validated as described in [31]), rabbit anti-β-actin (1:10,000, Merck KGaA, Darmstadt, Germany, A2066), and rabbit anti-tubulin β3 (1:1000, Synaptic System GmbH, Göttingen, Germany, 302302). Goat anti-rabbit peroxidase-conjugated antibodies (1:10,000, Merck KGaA, Darmstadt, Germany, A6154) were used as secondary antibodies, and the reaction was visualized using the SuperSignal^TM^ West Pico PLUS Chemiluminescent Substrate (ThermoFisher Scientific, Waltham, MA, USA). For densitometric analysis of WB, lanes for both loading controls (β-actin, tubulin β3) and protein of interest (Fbp2) were plotted and then peaks were labeled using the ImageJ software [32].

### 3.9. Statistical Analysis

Results are expressed as a mean and standard deviation. Data were checked for normality using the Shapiro–Wilk test, and for equality of variances using the F-test. For two groups of independent measurements with normal distribution the Student’s *t*-test was used, and for results deviating from normal distribution the Mann–Whitney’s *U*-test was used. For analysis of three or more independent cell culture conditions, the ANOVA test and the Tukey post hoc test were used. A probability of *p* < 0.05 was considered to be a significant difference. Fluorescence intensity normalized to control group is visualized with box plots. Center lines show the medians; box limits indicate the 25th and 75th percentiles as determined by R software (http://shiny.chemgrid.org/boxplotr/); whiskers extend 1.5 times the interquartile range from the 25th and 75th percentiles, and outliers are represented by dots.

## 4. Conclusions

**Figure 8:**
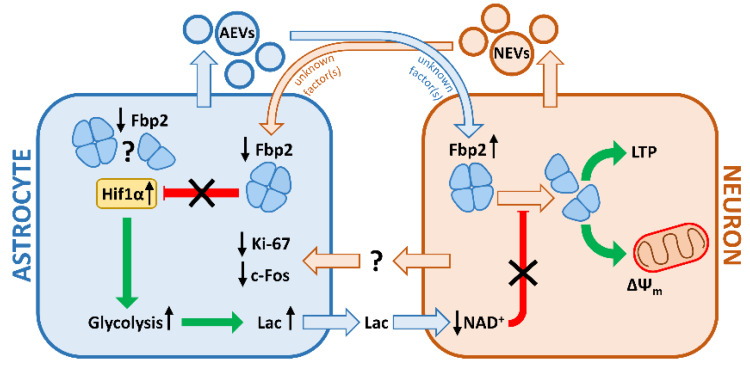
Overview of the key changes induced by the co-culturing of astrocytes and neurons (see text). AEVs—astrocytic extracellular vesicles; NEVs—neuronal extracellular vesicles; Lac—lactate; ΔΨm—mitochondrial membrane potential.

An overview of the key changes induced by the co-culturing of astrocytes and neurons is presented in Figure 8.

In murine hippocampal co-cultures of astrocytes and neurons, the level of Fbp2 protein is inversely modified by the intercellular crosstalk mediated by extracellular vesicle-packed factors.

In astrocytes, the reduction of the amount of Fbp2 correlates with the decrease kin Ki-67 and c-Fos—factors engaged in astrocyte proliferation and differentiation—and with the increase in Hif1α and, thus, intensification of glycolysis and lactate production. Astrocyte-to-neuron lactate shuttle reduces the level of Fbp2-tetramerizing NAD^+^ in neurons. At the same time, the astrocyte-derived factors increase the neuronal Fbp2 protein level. Together, this boosts protection of neuronal mitochondria membrane potential and prepares neurons for induction of NMDAR-dependent LTP.

## Supplementary Materials

The following are available online at https://www.mdpi.com/1422-0067/21/18/6903/s1.
