# Supplementary figures and images for: Expression of Fbp2, a Newly Discovered Constituent of Memory Formation Mechanisms, Is Regulated by Astrocyte–Neuron Crosstalk
Source: Int J Mol Sci. 2020 Sep 20;21(18):6903. doi: 10.3390/ijms21186903 (PMC7555702; doi:10.3390/ijms21186903)

A)

total ROS

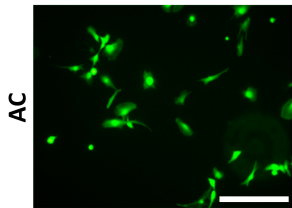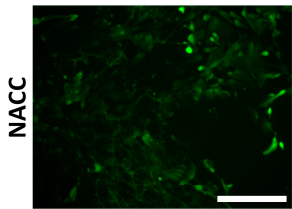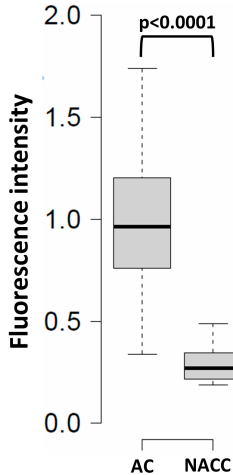

B)

mitochondrial ROS

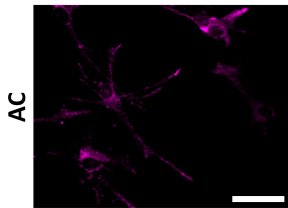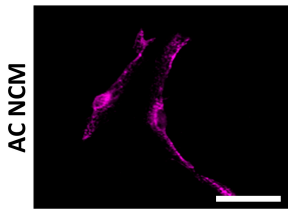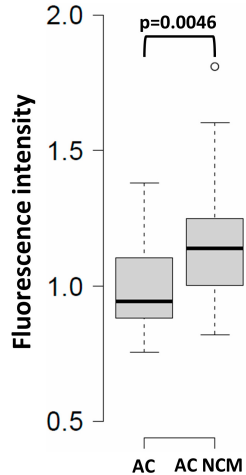

Supplement: Supplementary file 1 [file ijms-21-06903-s001.pdf]
